# Clinical suspicion and parathyroid carcinoma management

**DOI:** 10.1590/S1516-31802006000100009

**Published:** 2006-01-05

**Authors:** Fabio Luiz de Menezes Montenegro, Marcos Roberto Tavares, Marcelo Doria Durazzo, Claudio Roberto Cernea, Anói Castro Cordeiro, Alberto Rosseti Ferraz

**Keywords:** Hyperparathyroidism, Parathyroid glands, Parathyroid neoplasms, Parathyroid diseases, Parathyroidectomy, Hiperparatireoidismo, Glândulas paratireóides, Neoplasias das paratireóides, Paratireoidectomia, Doenças das paratireóides

## Abstract

**CONTEXT AND OBJECTIVE:**

Adequate management of parathyroid carcinoma apparently relates to the surgeon's ability to identify it at the first operation. The objective of this paper was to evaluate the role of clinical suspicion in the management of parathyroid carcinoma.

**DESIGN AND SETTING:**

Retrospective analysis of parathyroid carcinoma patients treated in Department of Head and Neck Surgery, Faculdade de Medicina da Universidade de São Paulo.

**METHODS:**

Cross-sectional study of 143 patients who underwent surgery from 1995 to 2000, due to hyperparathyroidism. These cases were reviewed to ascertain whether preoperative and intraoperative suspicion of parathyroid carcinoma were helpful during the operation, and which factors demonstrated the suspicion of cancer best.

**RESULTS:**

Among 66 patients with primary hyperparathyroidism there were four cases of parathyroid carcinoma (6.1%), and one case was found in secondary hyperparathyroidism (1.3%). Palpable nodules were found in five patients with primary hyperparathyroidism, four of them with parathyroid carcinoma. Preoperative levels of calcium in primary hyperparathyroidism with cancer patients varied from 12.0 mg/dl to 18.2 mg/dl. Two patients had gross macroscopic spread of the tumor to adjacent structures. Except for one patient, with extensive disease, tumors were resected *en bloc*. In secondary hyperparathyroidism, parathyroid carcinoma was found in a fifth mediastinal gland. One atypical adenoma was observed.

**CONCLUSIONS:**

High levels of calcium, palpable tumors and adherence to close structures are more common in parathyroid carcinoma. These clinical signs may be helpful for decision-making during parathyroid surgery.

## RESUMO

**CONTEXTO E OBJETIVO:**

A abordagem adequada do carcinoma de paratireóide parece relacionada à capacidade de identificação pelo cirurgião na primeira operação. O objetivo do estudo foi avaliar o papel da suspeita clínica pré-operatória na abordagem do carcinoma de paratireóide.

**TIPO DE ESTUDO E LOCAL:**

Análise retrospectiva realizada no Departamento de Cirurgia de Cabeça e Pescoço, da Faculdade de Medicina da Universidade de São Paulo, São Paulo, Brasil.

**MÉTODOS:**

De 1995 a 2000, 143 pacientes foram operados por hiperparatireoidismo. Esses casos foram revistos para verificar se a suspeita clínica pré e intra-operatória de carcinoma de paratireóide foi útil para a realização de operação mais extensa e quais fatores melhor se relacionaram com a suspeita de carcinoma.

**RESULTADOS:**

Entre 66 casos de hiperparatireoidismo primário, houve quatro casos de carcinoma de para-tireóide (6,1%), e um caso foi encontrado em hiperparatireoidismo secundário (1,3%). Nódulos palpáveis foram observados em cinco pacientes com hiperparatireoidismo primário, quatro deles com carcinoma de paratireóide. Os níveis pré-operatórios da calcemia nos casos de carcinoma de paratireóide de hiperparatireoidismo primário variaram entre 12,0 mg/dl e 18,2 mg/dl. Em dois pacientes, o tumor tinha invasão macroscópica de estruturas adjacentes. Com exceção de um caso, com doença extensa, houve ressecção completa em monobloco dos tumores. No hiperparatireoidismo secundário, o carcinoma de paratireóide foi identificado numa quinta glândula mediastinal. Houve um caso de adenoma atípico.

**CONCLUSÕES:**

O nível de calcemia muito elevado, o tumor palpável e a aderência a estruturas próximas são comuns no carcinoma de paratireóide. Esses sinais clínicos podem ser úteis na tomada de decisão nas operações sobre a glândula paratireóide.

## INTRODUCTION

Parathyroid carcinoma (PC) is considered to be a rare cause of hyperparathyroidism (HPT).^1^ Diagnosis of HPT due to PC is difficult, and 86% of the patients receive no intraoperative diagnosis of carcinoma.^1^

Although distinction between parathyroid adenoma and PC may be difficult even upon microscopic evaluation, the extent of the operation is very different in each disease. In PC, *en bloc* resection seems to be the best approach. Decision-making during parathyroid operation may be crucial to PC control.^2,3^

In some cases, clinical and surgical findings may raise suspicion of PC. The objective of this paper was to analyze the value of clinical suspicion in the management of PC, in a retrospective review of the cases treated in our service.

## PATIENTS AND METHODS

From 1995 to 2000, 143 patients were operated on for HPT at the Department of Head and Neck Surgery of Faculdade de Medicina da Universidade de São Paulo. Sixty-six patients presented with primary HPT and the remaining 77 cases were related to secondary HPT.

In the primary HPT cases, preoperative total calcium levels were compared with those of patients with PC in the present series (n = 4) and were also compared with the mean calcium levels of all patients treated for PC at the same institution since 1970, which were reported previously in another paper.^3^ This comparison was avoided in relation to secondary HPT because PC was observed in only one case.

The impact of clinical suspicion on the decision to perform en bloc resection was evaluated. Clinical suspicion was related preoperatively to high calcium levels (close to 14 mg/dl) and palpable neck masses. Intraoperative suspicion was related to invasion or adherence of the tumor to local structures.

Diagnosis of PC was based on pathological demonstration of a parathyroid tumor with invasion of capsule and blood vessels. Trabecular pattern, thick fibrous bands and the presence of mitosis were considered suspect, but not diagnostic in the absence of vascular or capsular invasion.

## RESULTS

Findings from primary HPT cases are presented in Table 1. In secondary HPT, parathyroid hyperplasia was observed in all cases.

**Table 1:** Diagnoses in primary hyperparathyroidism (1995-2000)

Diagnosis	n	%
Adenoma	47	71.2
Hyperplasia	12	18.2
Carcinoma	4	6.1
Double adenoma	1	1.5
Atypical adenoma	1	1.5
Parathyroid cyst	1	1.5
**Total**	**66**	**100**

Of the 143 patients, five had PC (3.5%). PC was more frequently observed in primary (6.1%) than in secondary HPT (1.3%).

Data from patients with PC in primary HPT are detailed in Table 2. Case 1 was included in a previous publication,^3^ but without details. The remaining three have not been reported before. Adherence to the thyroid was found in cases 2, 3 and 4. In case 1, invasion of the thyroid was suspected as the patient had a previous total thyroidectomy at another hospital. No lymph node metastasis was found in any cases.

**Table 2:** Patients with primary hyperparathyroidism and parathyroid carcinoma (PC)

	Case 1	Case 2	Case 3	Case 4
Age	64	22	50	29
Gender	Female	Female	Male	Male
Calcium (mg/dl)	12.0	14.9	13.4	18.2
Parathyroid hormone (PTH)*	7.2	19.2	11.6	20.8
Previous neck operation	Yes, related to PC	No	Yes, possibly unrelated to PC	No
Affected gland	Left inferior	Right superior	Left inferior	Left inferior
Extraparathyroidal extension	macroscopic	absent (capsular and vascular invasion)	macroscopic	microscopic
Type of resection	Partial	En bloc	En bloc (microscopic positive margin in review)	En bloc
Tumor size	4.0 × 2.0 × 2.0 cm + residual tumor in major vessels	3.0 × 2.0 × 2.0 cm	8.0 × 5.0 × 3.0 cm	2.8 × 2.8 × 1.8 cm
TNM†	T4 Nx M1 (recurrent + extensive local)	T2 N0 M0	T3 N0 M0	T3 N0 M0
Stage†	IV (lung metastasis)	II	IIIA	IIIA
Follow-up	Died of disease	Alive, NED‡ at 34 months	Rise in calcium / PTH at 18 months^§^	Alive, NED at 36 months

* *Times the normal upper limit for the method utilized;*

† *According to Shaha et al.^4^;*

‡ *NED = no evidence of disease;*

§ *Patient with extensive recurrence in the retropharyngeal space, died of disease at 61 months.*

Table 3 shows the results from comparative analysis of the demographic and clinical data of patients with PC and those with parathyroid adenoma.

**Table 3:** Characteristics of parathyroid carcinoma and adenoma cases in primary hyperparathyroidism

Characteristics	Carcinoma (n = 4)	Adenoma (n = 47)	p
Mean age (SEM)	41.2 (9.6)	52.2 (2.4)	0.20*
Female: male	1:1	3.3:1	0.27†
Palpable nodules n (%)	4 (100)	1 (2.1)	< 0.0001†
Mean calcium (SEM)	14.6 (1.3)	11.8 (0.2)	0.01‡

*SEM = standard error of the mean;*

* *Unpaired t test;*

† *Fisher's exact test;*

‡ *Mann-Whitney test.*

Including the data on the four cases here presented, 11 cases of PC in primary HPT have been treated in our service since 1970. The mean calcium level was 14.7 mg/dl, with standard error of the mean (SEM) of 0.7, which differed from the average value for adenoma in the present series (p = 0.001, unpaired t test with Welch's correction).

From 1995 to the present day in our hospital only one patient (a 31-year-old male) with secondary HPT had a PC. The clinical presentation was of recurrent HPT after four- gland parathyroidectomy, five years earlier. Preoperative calcium was 9.7 mg/dl and the parathyroid hormone (PTH) level was 2,475 pg/ml (normal range: 10-65 pg/ml). The tumor was a hard mass of 28 × 22 × 17 mm in a supernumerary mediastinal gland. Microscopy revealed areas of nuclear pleomorphism, thick fibrous bands, and blood vessel invasion with clusters of parathyroid cells attached to the vascular wall.

Among the primary HPT cases, one female patient aged 17 had a soft palpable parathyroid. The preoperative calcium levels fluctuated between 10.6 to 13.0 mg/dl and the PTH level was 1,010 pg/ml (normal range: 10-65 pg/ml). Upon surgical exploration, an enlarged inferior parathyroid was found, without adherence to local structures, and gland excision was easily performed. Some cellular atypia was present, but no microscopic signs of PC were observed. After more than five years of follow up, there is no evidence of HPT recurrence.

## DISCUSSION

Parathyroid carcinoma is still a diagnostic and therapeutic challenge.^4^ Isolated excision of a PC is frequently followed by recurrence, and extended resection including the thyroid lobe offers a more favorable prognosis. In the present series, all patients with primary HPT operated on with PC had preoperative suspicion of malignancy, and complete resection with margins was the plan.

High calcium levels may be the first clue to PC. Despite the statistically significant difference in mean calcium level in patients with PC in this study, calcium should be analyzed in the whole context, as some patients with parathyroid adenoma would have high calcium levels.

High PTH levels have been reported as a possible indicator for PC.^2^ In the present authors’ experience, some patients with adenoma or secondary HPT may present very high PTH levels without any evidence of malignancy (unpublished data).

Palpable cervical masses are usually present in half of patients with PC.^3^ Parathyroid adenoma is seldom palpable. In the present series, palpable parathyroid was found in less than 3% of the patients with adenoma.

The finding of PC in 6.1% of the cases of primary HPT is comparable to findings in a Japanese^2^ and a recent Italian^5^ study with an incidence of 5%. Apart from the fact that more complicated cases are selectively referred to our hospital, few patients seem to be diagnosed with asymptomatic or mild HPT in this country.

Variable biological features are probably found in PC,^1-3^ and this leads to different clinical behavior and disease progression. The recent proposal of a staging system for PC^4^ is laudable, but based on the experience here reported, a distinction between microscopic or macroscopic extraparathyroidal extensions in T3 tumors may need to be considered.

## CONCLUSIONS

High calcium levels and a palpable cervical mass are indicative of PC, and these characteristics are less frequently present under benign conditions.
